# Interactions between the quality control ubiquitin ligase CHIP and ubiquitin conjugating enzymes

**DOI:** 10.1186/1472-6807-8-26

**Published:** 2008-05-16

**Authors:** Zhen Xu, Ekta Kohli, Karl I Devlin, Michael Bold, Jay C Nix, Saurav Misra

**Affiliations:** 1 Department of Molecular Cardiology, Lerner Research Institute, The Cleveland Clinic, Cleveland, Ohio, USA; 2 Molecular Biology Consortium, Advanced Light Source, Lawrence Berkeley National Laboratory, Berkeley, California, USA

## Abstract

**Background:**

Ubiquitin (E3) ligases interact with specific ubiquitin conjugating (E2) enzymes to ubiquitinate particular substrate proteins. As the combination of E2 and E3 dictates the type and biological consequence of ubiquitination, it is important to understand the basis of specificity in E2:E3 interactions. The E3 ligase CHIP interacts with Hsp70 and Hsp90 and ubiquitinates client proteins that are chaperoned by these heat shock proteins. CHIP interacts with two types of E2 enzymes, UbcH5 and Ubc13-Uev1a. It is unclear, however, why CHIP binds these E2 enzymes rather than others, and whether CHIP interacts preferentially with UbcH5 or Ubc13-Uev1a, which form different types of polyubiquitin chains.

**Results:**

The 2.9 Å crystal structure of the CHIP U-box domain complexed with UbcH5a shows that CHIP binds to UbcH5 and Ubc13 through similar specificity determinants, including a key S-P-A motif on the E2 enzymes. The determinants make different relative contributions to the overall interactions between CHIP and the two E2 enzymes. CHIP undergoes auto-ubiquitination by UbcH5 but not by Ubc13-Uev1a. Instead, CHIP drives the formation of unanchored polyubiquitin by Ubc13-Uev1a. CHIP also interacts productively with the class III E2 enzyme Ube2e2, in which the UbcH5- and Ubc13-binding specificity determinants are highly conserved.

**Conclusion:**

The CHIP:UbcH5a structure emphasizes the importance of specificity determinants located on the long loops and central helix of the CHIP U-box, and on the N-terminal helix and loops L4 and L7 of its cognate E2 enzymes. The S-P-A motif and other specificity determinants define the set of cognate E2 enzymes for CHIP, which likely includes several Class III E2 enzymes. CHIP's interactions with UbcH5, Ube2e2 and Ubc13-Uev1a are consistent with the notion that Ubc13-Uev1a may work sequentially with other E2 enzymes to carry out K63-linked polyubiquitination of CHIP substrates.

## Background

Ubiquitination is a key posttranslational modification that is involved in most aspects of cellular homeostasis, signalling and regulation. In ubiquitination, sequential action of E1 (ubiquitin-activating), E2 (ubiquitin-conjugating) and E3 (ubiquitin ligase) proteins act sequentially to promote attachment of the 76 amino-acid polypeptide ubiquitin to a substrate protein[1,2]. Ubiquitin is attached to the substrate through an isopeptide bond between the C-terminus of ubiquitin and the ε-amino group of a substrate lysine. E1 enzymes transfer ubiquitin to the active site cysteine of an E2 enzyme [3]. E2 enzymes, in turn, bind to E3 ligases, which contain either HECT domains or a member of the structurally similar RING, PHD-like and U-box domain superfamily [4-6]. E3 ligases also contain other domains that directly or indirectly bind substrate proteins. E3 ligases thus bring together ubiquitin-conjugated E2 enzymes and particular substrates, and also catalyze ubiquitin transfer to substrate lysines [4]. In the absence of substrates, some E3 ligases can promote the attachment of ubiquitin to one of their own lysines, in a process termed "autoubiquitination" [7-9].

Ubiquitination can result in the addition of a single ubiquitin (monoubiquitination) but also in the addition of successive ubiquitins to the first (polyubiquitination) [10]. Successive ubiquitins are linked through one of the seven ubiquitin surface lysines. Modification by a K48-linked polyubiquitin chain targets the substrate for proteasomal degradation, the intracellular fate commonly associated with ubiquitination [11]. Other polyubiquitin chains and monoubiquitin play roles in trafficking, DNA repair, signal transduction, transcriptional regulation and other processes [12,13].

E2 enzymes exhibit high similarity in sequence and structure [14]. There are several dozen E2 enzymes and hundreds of E3 ligases, which carry out ubiquitination in different combinations. As the identity of the E2 enzyme recruited by an E3 ligase to a particular substrate crucially influences the type and consequences of the ubiquitination, it is important to characterize the structural and biochemical parameters that govern the specificity of E2:E3 interactions.

The C-terminus of Hsp70 interacting protein (CHIP) is a homodimeric ubiquitin ligase that links chaperone function with the degradation of misfolded or unstable proteins [15,16]. As such, CHIP plays a key role in protein quality control, serving as a disposal mechanism for conformationally intractable proteins that might otherwise overwhelm the chaperone system. CHIP binds to C-terminal motifs found in the chaperones Hsp70 and Hsp90. CHIP has three domains: an N-terminal TPR (tetratricopeptide repeat) domain through which it binds Hsp70 or Hsp90, a middle helical domain that mediates CHIP dimerization [17], and a C-terminal U-box domain that binds specific E2 enzymes [5]. By recruiting a cognate E2 enzyme, CHIP promotes ubiquitination of numerous labile client proteins that are chaperoned by Hsp70 or Hsp90 [18-20]. These clients include proto-oncogene products, kinases, nuclear hormone receptors, and aggregation-prone proteins, such as α-synuclein and tau, that are involved in neurodegenerative diseases [15,16,21].

Two E2 enzymes, UbcH5 and Ubc13, have been identified as cognates for CHIP [18,20,22]. "UbcH5" refers collectively to three highly homologous E2 enzymes, UbcH5a, b and c (88% identical, 95% similar). While the UbcH5 enzymes were previously thought to carry out K48-linked ubiquitination exclusively, recent studies have shown that they can form mixed-linkage polyubiquitin chains *in vitro *with several E3 ligases, including CHIP [23]. Recently, Pearl and coworkers showed that CHIP also interacts with Ubc13-Uev1a [22,24], a heterodimeric E2 enzyme that exclusively forms K63-linked chains [25]. K63-linked chains are primarily associated with trafficking and substrate regulation (through the mediation of new protein-protein interactions) rather than degradation [26]. The crystal structure of Ubc13-Uev1a bound to the U-box of CHIP shows that Ubc13 interacts with the CHIP U-box, while neither subunit of the Ubc13-Uev1a heterodimer contacts the CHIP-TPR or dimerization domains [22].

Interaction between CHIP and these two different E2 enzymes likely results in different types of polyubiquitination and different biological outcomes. As UbcH5 and Ubc13 are similar in sequence and structure, it is unclear whether and how CHIP distinguishes between these E2 enzymes. To obtain additional insights into CHIP specificity, we solved the crystal structure of Zebrafish UbcH5a bound to the CHIP U-box domain. Despite an overall structural similarity, CHIP exhibits differences in the details of individual interactions in the UbcH5a and Ubc13-Uev1a complexes, as well as in the ability of the respective enzymes to carry out CHIP autoubiquitination (self-ubiquitination of CHIP in the absence of Hsp70 or Hsp90). The structures of the two complexes allow us to further define a set of specificity determinants required for interaction with CHIP. Based on these determinants, we identify several class III E2 enzymes as an additional, third group of cognate E2 enzymes for CHIP. As the first E3 ligase to be structurally characterized in complex with two different E2 enzymes, CHIP may serve as a useful model for understanding the basis of specificity in other E2:E3 interactions.

## Results

### Structure of the CHIP U-box: UbcH5a complex

We previously investigated the interaction between CHIP and UbcH5b using NMR and site-directed mutagenesis [27]. This allowed us to identify respective binding interfaces on each protein and to propose a model for the CHIP U-box:UbcH5b complex. The model, however, was ambiguous with respect to specific interactions mediated by particular main-chain and side-chain groups, especially those involving the prominent L4 and L7 loops on the E2 enzyme. To obtain a more accurate understanding of how CHIP binds to members of the UbcH5 family, we screened full-length human and Zebrafish CHIP, as well as their respective U-box domains, for cocrystallization with human and Zebrafish UbcH5a, UbcH5b and UbcH5c. We crystallized a complex of the Zebrafish CHIP U-box and UbcH5a and solved its structure to 2.9 Å resolution by molecular replacement (see Methods). Statistics for the structure solution are listed in Table 1.

**Table 1:** Crystallographic and Refinement Statistics

	**CHIP U-box: UbcH5a complex**
**Data Collection**	
Space group	P2_1_2_1_2_1_
Unit cell	
a, b, c (Å)	79.0, 93.4, 144.0
α,β,γ (°)	90, 90, 90
No. molecules/asymmetric unit	2 CHIP, 2 UbcH5a
Resolution (Å)	55.00-2.90 (3.00-2.90)^a^
Unique reflections	24283
Completeness (%)	99.9 (100)
Redundancy	3.40 (3.42)
I/σ	8.5 (1.6)
R_merge_(%)^b^	8.0 (46.6)
	
**Refinement**	
Resolution (Å)	53.2-2.90 (3.08-2.90)
R_work_(%)^c^	24.0
R_free_(%)^d^	27.2
Protein atoms	3518
Waters	4
Other atoms	3
Average B factor (Å^2^)	65.0
r.m.s.d. – bond lengths (Å)	0.007
r.m.s.d. – bond angles (°)	1.4
r.m.s.d. – dihedrals (°)	23.4
r.m.s.d. – improper angles (°)	1.1
Ramachandran plot (%)	
Most favored and allowed	97.6
Generously Allowed	2.4
Disallowed	0.0
	
**PDB ID code**	2 OXQ

^a^Statistics for the highest resolution shell are shown in parentheses.

^b^R_merge _= Σ|I_i_-<I_i_>|/Σ|I_i_|

^c^R_work _= Σ_h_||F_obs_(h)|-k|F_calc_(h)||/Σ_h_F_obs_(h)

^d^R_free _is the R factor calculated for 5% of reflections, excluded from refinement

**Figure 1:**
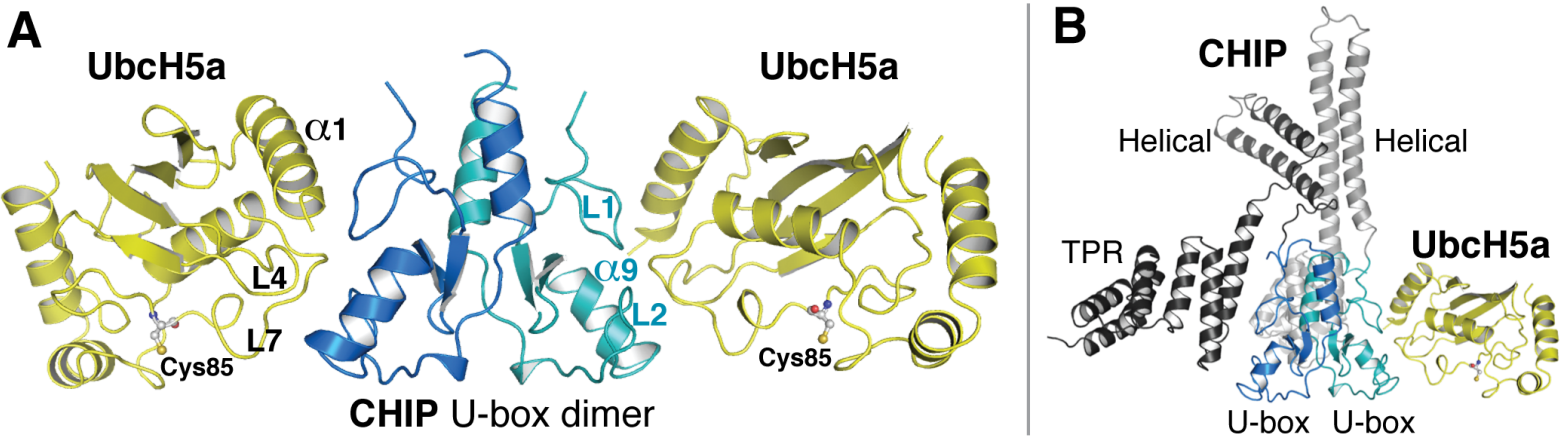
**Structure of the CHIP:UbcH5a complex**. **A**. Asymmetric unit of the crystallized zebrafish CHIP U-box: UbcH5a complex, showing the U-box dimer (blue) with two bound UbcH5a molecules (yellow). Secondary structure elements that mediate the interaction between the U-boxes and UbcH5a are labelled in blue or black for the U-boxes and UbcH5a respectively. Active site cysteines of the UbcH5a molecules are shown in ball-and-stick representation and labelled. **B**. Model of the CHIP:UbcH5a complex, CHIP, built by superimposing the U-box dimer and one UbcH5a molecule from the structure shown in panel A onto the crystal structure of murine CHIP, PDB:2C2L [22]. The TPR and helical domains of the two CHIP monomers are coloured light and dark grey. One TPR domain is behind the rest of the molecule and is left unlabelled for clarity.

The crystal asymmetric unit contains a back-to-back U-box dimer, with each U-box bound to an UbcH5a molecule (Figure 1A). The U-box conformation and dimerization mode are essentially identical to those in previously described structures of free CHIP (rms_d _< 0.4 Å), emphasizing that the U-box undergoes negligible conformational change upon binding to an E2 enzyme. The CHIP U-box structure is characteristic of the RING/PHD-like/U-box fold superfamily [4-6]. It consists of a small β-sheet, a central α-helix (termed CHIP-α9, as the other two domains of CHIP contain eight other alpha helices) and a C-terminal helix (CHIP-α10) that participates in U-box dimerization. The U-box also has two prominent loops, CHIP-L1 and CHIP-L2, which, together with CHIP-α9, comprise the interaction site for E2 enzymes [22].

While several structures of UbcH5 family E2 enzymes have been solved previously [28,29], this is the first to be solved in complex with an E3 ligase domain. UbcH5a adopts the characteristic fold exhibited by all E2 enzyme catalytic cores, consisting of a central β-sheet flanked by 4 helices. The N-terminal α-helix (UbcH5a-α1) and two prominent loops, UbcH5a-L4 and UbcH5a-L7, comprise most of one side the protein and interact with the CHIP U-box (Figure 1A), contacting a hydrophobic groove formed by the interface of CHIP-L1, CHIP-L2 and CHIP-α9. The interface buries ~700 Å^2 ^of solvent-accessible surface area. The overall CHIP U-box:UbcH5a complex resembles the structures of the CHIP U-box: Ubc13-Uev1a complex [22] and the complex of the c-Cbl RING domain with the E2 enzyme UbcH7 [30].

Full-length CHIP is an asymmetric homodimer, in which the conformation of one monomer is such that its TPR domain occludes the U-box domain of the same monomer; the CHIP dimer thus contains only one E2 binding site [22]. We superimposed the U-box:UbcH5a complex onto the structure of full-length mouse CHIP (Figure 1B). The model of the CHIP:UbcH5 complex reemphasizes that E2 interactions with CHIP are mediated only through the U-box domain, while no contacts are made between the E2 and the helical or TPR domains of the CHIP dimer.

### Analysis of interface between CHIP U-box and UbcH5a

**Figure 2:**
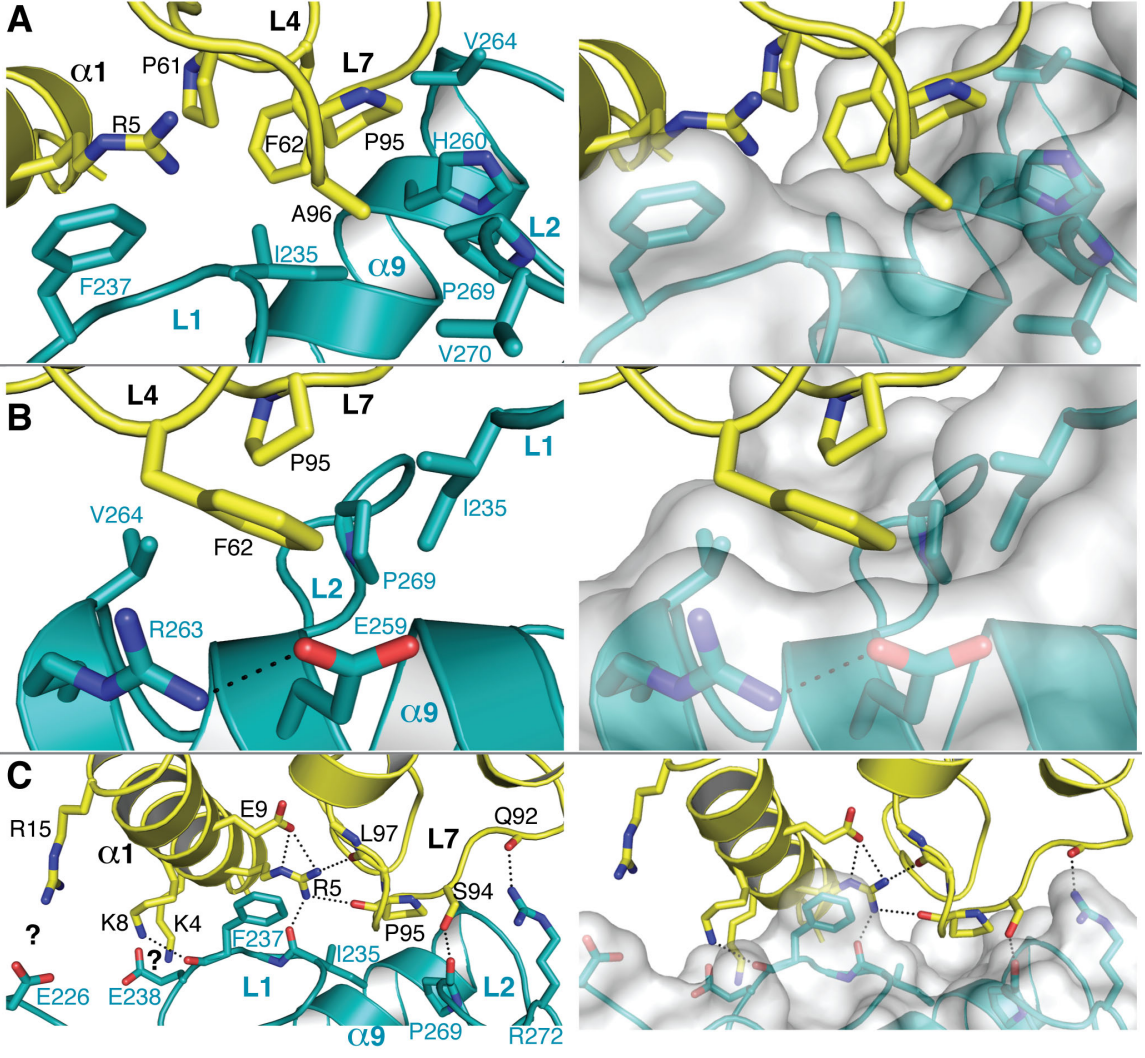
**Details of the interaction between UbcH5a and the CHIP U-box**. The view in panel C is zoomed out from that in panels A and B. For clarity, only side-chain or main-chain atoms of some residues are shown. Panels on the right show molecular surface representations of the CHIP U-box. **A**. Closeup of the hydrophobic packing between UbcH5a (yellow) and CHIP (blue). The interacting secondary structure elements and residues are labelled in black (UbcH5a) or blue (CHIP). **B**. "Back" view of the complex emphasizing the hydrophobic packing between UbcH5a-loop L2 (the F62 sidechain) and the CHIP U-box. **C**. Polar interactions between UbcH5a and CHIP. Likely hydrogen bonds or salt bridges are shown as dotted lines. Acidic and basic side chains that are in proximity but not close enough or in the correct geometry to form salt bridges are marked with question marks.

Key interactions between the CHIP U-box and UbcH5a are shown in Figure 2. (For compatibility with our *in vitro *ubiquitination assays, which were carried out using human rather than zebrafish CHIP, we refer to all CHIP residues according to the numbering in the human protein). The interactions are arranged around a central hydrophobic patch, in which the sidechain of UbcH5a-F62 protrudes into a concave surface formed by aliphatic portions of sidechains from both loops and helix α9 of the U-box (Figure 2A). The phenyl ring of UbcH5a-F62 also stacks against the guanidinium group of CHIP-R263, which is held in position by a salt bridge with CHIP-E259 (Figure 2B). These two large sidechains thus form the "back wall" of a primarily hydrophobic dimple in the CHIP U-box surface, into which UbcH5a protrudes. UbcH5a-F62 is flanked by other aliphatic residues (UbcH5a-P61, P95, A96), which contribute to the hydrophobic interaction area. An additional interaction is formed by planar stacking of the aromatic sidechain of CHIP-F237 against the guanidinium group [31] of UbcH5a-R5, which leaves the arginine sidechain free to participate in an extensive *intra*-UbcH5a polar network (Figure 2A, C).

The abovementioned interactions are flanked by polar interactions (Figure 2C), among which hydrogen bonds between the sidechains of UbcH5a-R5, UbcH5a-S94, and CHIP-R272 with complementary main chain groups are prominent. Several additional interactions, such as the one between UbcH5a-K8 and the CHIP-F237 carbonyl oxygen, are likely to be weak, as they are present in one of the U-box: UbcH5a complexes in the crystal asymmetric unit but not in the other. In addition, there are several charged residues on UbcH5-α1 and CHIP-L1 (labelled with question marks in Figure 2B) that are near enough to each other to potentially engage in salt bridge interactions. The relevant sidechains, however, exhibit suboptimal interaction geometries or actually point away from their putative partner residues, suggesting that the corresponding salt bridges are weak or disfavoured.

In the absence of Hsp70/Hsp90 and a chaperone-bound client, CHIP itself is ubiquitinated by UbcH5 family E2 enzymes [20,24]. CHIP autoubiquitination is an effective reporter of a productive interaction between CHIP and UbcH5 enzymes. We previously mutagenized the surface of the CHIP U-box to identify residues important for its interaction with UbcH5 family E2 enzymes, as gauged by *in vitro *CHIP autoubiquitination assays and western blotting with anti-ubiquitin antibody [27]. We have now carried out ELISA-based assays (see Methods) in Nickel-NTA coated microplates to quantitate autoubiquitination of His-tagged human CHIP by human UbcH5b (which is identical to UbcH5a in the CHIP-interacting region). Our results confirm the key importance of the hydrophobic CHIP residues lining the intersection of the L1 and L2 loops and helix α9, including CHIP-I235, H260 and V264, as CHIP is autoubiquitinated poorly or not at all by the corresponding alanine mutants (see below). Similarly, flanking interactions mediated by F237 and R272 are crucial for productive CHIP:UbcH5 interactions, as the corresponding alanine mutants also eliminate CHIP autoubiquitination (see below).

### Comparison between CHIP:UbcH5a and CHIP:Ubc13-Uev1a complexes

**Figure 3:**
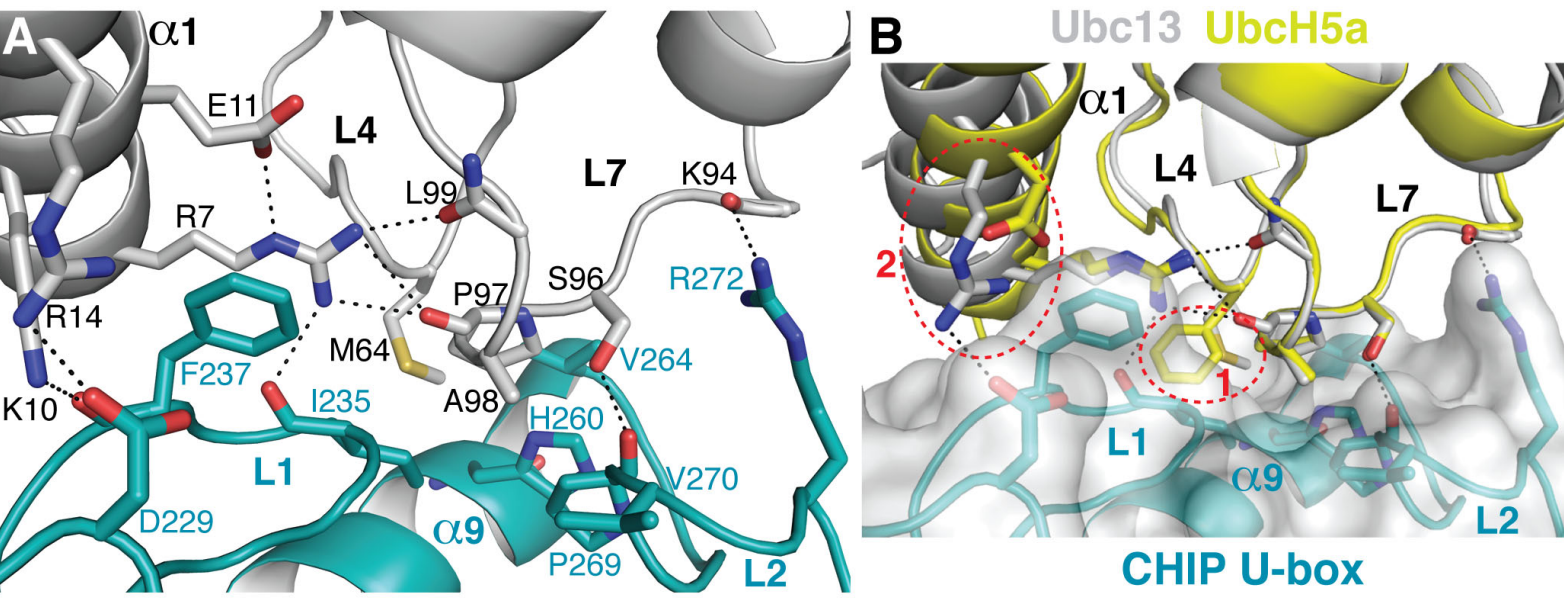
**Interaction between Ubc13 and the CHIP U-box**. Only the main-chain or side-chain atoms of some residues are shown for clarity. Hydrogen bonds and salt bridges are shown as dotted lines. **A**. Interactions between Ubc13 (white) and the CHIP U-box (cyan), from PDB:2C2V [22]. **B**. Superimposition of the UbcH5a:CHIP U-box and Ubc13:CHIP U-box complexes (UbcH5a in yellow, Ubc13 in white). As the conformations of the CHIP U-box backbone and interface residues are almost identical between the structures, only the U-box from the Ubc13:CHIP complex is shown. Several key interacting residues are shown explicitly. The red dashed circles indicate two interaction sites that differ between the two E2 enzymes.

Many of the interactions shown in Figure 2 are also conserved in the complex between CHIP and Ubc13-Uev1a (Figure 3A). Superimposition of the UbcH5a:CHIP U-box and Ubc13:CHIP U-box complexes (Figure 3B) suggests that the interaction surfaces in the two complexes are similar, though there are several detailed differences that may influence the relative strengths of the interactions. The α1 helices of the two E2 enzymes are the most divergent in terms of overall structure, but this difference does not translate into large differences in the locations of the CHIP-interacting residues, such as UbcH5a-R5/Ubc13-R7, on these helices (Figure 3B).

**Figure 4:**
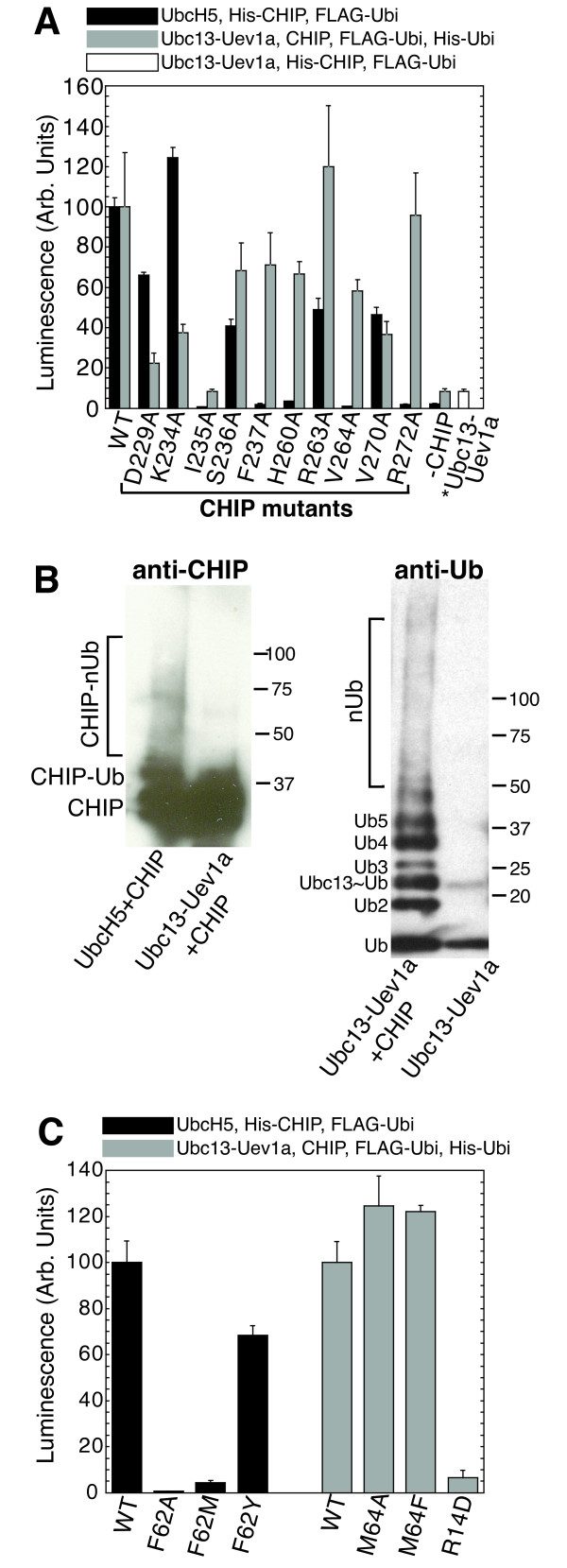
**Mutagenesis of CHIP:E2 interaction surfaces**. **A**. *In vitro *ubiquitination measurements of CHIP with UbcH5 or Ubc13-Uev1a carried out using an ELISA-based assay. Wild-type or mutant His-CHIP bound to Nickel-NTA coated microplates was autoubiquitinated with FLAG-Ubiquitin in the presence of UbcH5b (dark grey bars). Mixed His- and Flag-tagged polyubiquitin chains were formed in Nickel-NTA coated microplates in the presence of untagged CHIP and Ubc13-Uev1a (light grey bars). The white bar ("*Ubc13-Uev1a") represents (minimal) autoubiquitination of His-CHIP by FLAG-Ubiquitin in the presence of Ubc13-Uev1a. "-CHIP" designates negative controls carried out with no CHIP in the reactions. Data are in arbitrary units and were normalized by the values measured for the respective E2 enzymes with wild-type CHIP. **B**. Autoubiquitination of CHIP in the presence of UbcH5 but not Ubc13-Uev1a, probed by western blotting. Anti-ubiquitin staining confirms the formation of polyubiquitin chains by Ubc13-Uev1a in a CHIP-dependent manner. Anti-CHIP staining shows that a fraction of CHIP is ubiquitinated (higher-molecular weight bands) in the presence of UbcH5 but not Ubc13-Uev1a. **C**. Ubiquitination measurements of wild-type CHIP with wild-type and mutant UbcH5 or Ubc13-Uev1a, as described for panel A.

Since UbcH5 and Ubc13-Uev1a form different types of polyubiquitin chains [23,25], we investigated whether mutations that prevented autoubiquitination of CHIP by UbcH5 have similar effects on Ubc13-Uev1a. We initially tried to detect autoubiquitination of His-CHIP by Ubc13-Uev1a in Nickel-NTA plates as described above for UbcH5, but observed no CHIP autoubiquitination above background (Figure 4A, column marked "*Ubc13-Uev1a"). Several previous studies have suggested that CHIP catalyzes the formation of unanchored K63-linked polyubiquitin chains by Ubc13-Uev1a, but that these chains are not conjugated to CHIP itself [24,30]. We reconfirmed this by western blotting of *in vitro *ubiquitination assays. CHIP promoted the formation of polyubiquitin chains by Ubc13-Uev1a, detected using anti-Ubiquitin antibody. No higher-molecular weight species indicative of polyubiquitinated CHIP, however, were detectable by western blotting with anti-CHIP (Figure 4B).

Our initial ELISA assay selected exclusively for His-tagged CHIP conjugated to Flag-ubiquitin. We therefore carried out a second assay using a mixture of His- and Flag-tagged ubiquitins to detect polyubiquitin chains that were not conjugated to CHIP. Our results indeed confirm that CHIP catalyzes Ubc13-Uev1a-mediated formation of unanchored, mixed Flag- and His-tagged ubiquitin chains that were readily quantified (Figure 4A). However, despite the structural similarity between the CHIP:UbcH5a and CHIP:Ubc13 interfaces (Figure 3B), several mutations that do not catalyze ubiquitination with UbcH5b are substantially active with Ubc13-Uev1a. Of the mutants tested, only CHIP-I235A is inactive with both E2 enzymes (Figure 4A).

To explore why Ubc13-Uev1a is less sensitive to many of the mutations that eliminate productive interactions between UbcH5 and CHIP, we investigated the two most obvious differences in the CHIP:UbcH5a and CHIP:Ubc13 interfaces (Figure 3B). First, Ubc13-M64 is located in the same position as UbcH5-F62. Second, a salt bridge between Ubc13-R14 and CHIP-D229 is not conserved in the CHIP:UbcH5a complex; instead, UbcH5a-D12 is the equivalent of Ubc13-R14. We mutagenized these sites to investigate their importance for CHIP:E2 interactions. Surprisingly, Ubc13 is largely insensitive to mutations at the M64 position. In contrast, UbcH5a does not tolerate a methionine at the F62 position (Figure 4C). The interaction between R14 and CHIP-D229 also contributes to binding between Ubc13 and CHIP, as Ubc13-R14D is inactive in polyubiquitin chain formation (Figure 4C). Similarly, the CHIP-D229A mutant is less active in polyubiquitin chain formation by Ubc13-Uev1a than any CHIP mutants other than CHIP-I235A (Figure 4A). In contrast, the D229A mutation has little effect on CHIP autoubiquitination by UbcH5b.

Thus, M64 is not required to anchor the hydrophobic interface between Ubc13 and CHIP, possibly because Ubc13 has at an additional compensating salt bridge to buttress its interaction with CHIP. Indeed, the hydrophobic interface is less important overall for the Ubc13:CHIP interaction, and the CHIP-H260A and CHIP-V264A mutants also exhibited partial activity with Ubc13-Uev1a. These data suggest that the detailed binding energetics differ between the CHIP:UbcH5a and CHIP:Ubc13-Uev1a complexes, in terms of the relative importance of individual interactions for overall binding.

### Influence of S-P-A motif in E2 enzymes on interaction with CHIP

One of the points of most intimate contacts between CHIP and UbcH5a or Ubc13-Uev1a is located at the interface of the CHIP U-box and loop L7 of the E2 enzymes (Figure 2). On this loop, the motif S-P-A (residues 94–96 of UbcH5a or residues 96–98 of Ubc13) makes a hydrogen bond with the carbonyl group of CHIP-P269 and van der Waals contacts with CHIP-H260, V264 and V270. As previously noted by Pearl and coworkers, the first position within this motif is unlikely to tolerate bulkier sidechains than serine, as these would clash with the surface of the CHIP U-box [22]. Previous studies indeed suggest that E2 enzymes that do not contain the S-P-A motif are not cognate E2 enzymes for CHIP (Table 2).

**Table 2:** S-P-A motif conservation in E2 enzymes

**E2 enzyme**	**Loop 7 Sequence**	**Interacts w. CHIP?**	**Reference**
Ubc1	DQWAAA	N	[20]
UbcH2	QTWTAL	N	[20]
Ubc3	ERWN**P**T	N	[18, 20]
UbcH7	ENWK**PA**	N	[18, 20]
UbcH8	ENWK**P**C	N	[18]
UbcH5a-c	SQW**SPA**	Y	[20]
Ubc13	DKW**SPA**	Y	[22, 24]
Ube2e1-3	DNW**SPA**	Y	*

Residues on loop 7 of selected E2 enzymes, corresponding to the positions of the S-P-A motifs in UbcH5 and Ubc13.

*Reported in this study.

**Figure 5:**
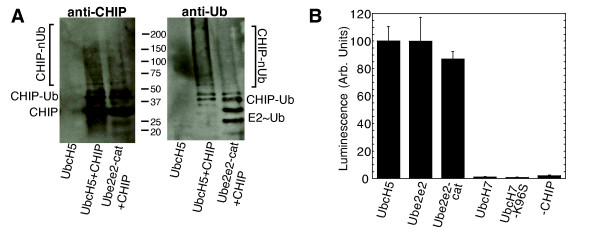
**CHIP interactions with Ube2e2 and UbcH7**. **A**. Autoubiquitination of CHIP in the presence of Ube2e2 catalytic domain (Ube2e2-cat) probed by western blotting. Anti-ubiquitin staining confirms the formation of polyubiquitin chains. Anti-CHIP staining shows that a fraction of CHIP is modified by mono- and polyubiquitin, migrating as higher-molecular weight bands. **B**. *In vitro *ubiquitination measurements of CHIP with Ube2e2 and UbcH7 carried out using the ELISA-based assay. His-CHIP bound to Nickel-NTA coated microplates was autoubiquitinated with FLAG-Ubiquitin in the presence of E2 enzymes as shown. UbcH5b was used as the positive control; a reaction containing UbcH5b but no His-CHIP was used as a negative control ("-CHIP"). Counts were normalized by the value measured for the positive control.

We examined the sequences of human E2 enzymes for those containing S-P-A motifs on loop L7, and with no residues at other positions that would obviously prevent interaction with the CHIP U-box. Several Class III E2 enzymes, Ube2e1, Ube2e2 and Ube3e3, fulfilled these criteria. Class III E2 enzymes, by definition, contain a N-terminal sequences that precede an E2 catalytic domain. The Ube2e1-3 enzymes, which contain 40–50 residue N-terminal extensions [32], undergo nuclear translocation after being charged with ubiquitin from E1 [33]. While their N-terminal regions differ, their catalytic domains are highly homologous (95% identical, 99% similar). We tested full-length Ube2e2 as well as its catalytic domain (Ube2e2-cat) for polyubiquitin chain formation in the presence of CHIP by western blotting and by the ELISA-based ubiquitination assay (Figure 5). Like UbcH5, Ube2e2cat autoubiquitinates CHIP. Based on the very high homology between the Ube2e1-3 catalytic domains, it is likely that Ube2e1 and Ube2e3 are also cognate E2 enzymes for CHIP.

We used another E2 enzyme, UbcH7, to investigate whether the S-P-A motif is sufficient for conferring compatibility with CHIP. UbcH7 contains a Lysine at the position equivalent to the Serine of the S-P-A motif. We tested both wild-type UbcH7 and UbcH7-K96S for CHIP autoubiquitination (Figure 5B). Surprisingly, neither carried out polyubiquitination with CHIP, suggesting that the presence of an S-P-A motif in an E2 enzyme is necessary but not sufficient for productive interaction with CHIP.

## Discussion

Understanding the factors governing selectivity between E2 enzymes and E3 ligases is important for several purposes: 1) identifying or predicting which E2:E3 pairs govern a particular ubiquitin-linked pathway; 2) engineering E2:E3 pairs that can be used to identify substrates targeted by a particular E2:E3 combination; 3) rationalizing the functional diversity that characterizes many intracellular ubiquitination pathways. While there are numerous structures of isolated E2 enzymes and E3 ligases, there are relatively few structures of E2:E3 complexes. The structures of two RING/U-box domains in complex with E2 enzymes have been solved previously: the Cbl-RING domain with UbcH7 [30] and the CHIP U-box domains with Ubc13-Uev1a [22]. In addition, a complex between the CNOT4 RING domain and UbcH5b was modelled based on a comprehensive series of NMR and mutagenesis experiments [34].

We add to the above list the structure of the CHIP U-box: UbcH5a complex. The CHIP U-box is the first ubiquitin ligase domain to be structurally characterized with two different E2 enzymes, shedding additional light on key specificity determinants that govern E2:E3 interactions. A number of determinants are conserved among all the structurally characterized E2:E3 complexes, partly reflecting sequence homology among the ligase-interacting portions of E2 enzymes [14]. Table 3 lists key interactions found in the complexes, grouping those that are located in equivalent positions.

**Table 3:** Key interactions in structurally characterized E2:E3 complexes

	**E2:E3 Complex [PDB:ID Code]**			
**Interaction E2: E3**	**Cbl:UbcH7 [PDB:1FBV]**	**CNOT4:UbcH5 [PDB:1UR6]**	**CHIP:UbcH5 [PDB:2OXQ]**	**CHIP:Ubc13 [PDB:2C2V]**
**E2-α1: E3-L1**				
Arg:mainchain	R6:K382O precedes I383)	R5:P15O precedes L16)	R5:I235O	R7:I235O
Arg:sidechain		R5:M18	R5:F237 (stacking)	R7:F237 (stacking)
salt bridges		K4:E19/K4:E13 K8:E13		R14:D229
sidechain: mainchain			K8:F237O	K10:F237O
				
**E2-L4/L7: E3-α/L1/L2**				
hydrophobic	P62/F63/P97/A98:I383/W408/P417/F418	P61/F62/P95/A96:L16/I45/E49/L52/P54	P61/F62/P95/A96:I235/H260/V264/P269/V270	P63/M64/P97/A98:I235/H260/R263/V264/P269/V270
E2-L4:E3-α polar		F62:R44 (stacking) Q92: E49	F62:R263 (stacking)	
E2-L4:E3-α salt bridges		D59:R44 K68: D48/E49		
				
**E2-L7: E3-L2 polar**				
S-P-A:mainchain	A98N:P417O K96:F418O	S96:P54O	S94:P269O	S96:P269O
mainchain:Arg	E93O:R420	Q92O:R57	Q92O:R272	K94O:R272

Key interactions observed in structures of E2:E3 complexes [22, 30, 34]. The CNOT4:UbcH5 complex structure was not solved explicitly but modeled based on NMR structures of CNOT4 and UbcH5 and on experimental restraints derived from NMR data and mutagenesis [34].

The central hydrophobic interface, formed between aliphatic groups from loops L4 and L7 of the E2 enzyme and a cluster of aliphatic groups from both structured loops and the central helix of the RING/U-box domains, is likely present in all complexes formed between E2 enzymes and RING/PHD-like/U-box ligases. A ring of primarily hydrophobic residues from the L1 and L2 loops and the central helix of the RING/U-box ligase domains define the walls of a concavity into which the tip of E2-L4 protrudes. These include a hydrophobic residue on loop L1 (such as CHIP-I216) that is an isoleucine, leucine or valine in essentially all RING/U-box domains.

The conformations of the interacting loops on both partners are strongly influenced by the positions of highly conserved proline residues, which interact directly with the rim of the hydrophobic concavity on the ligase domain. These prolines also help to position other E2 residues such as the large hydrophobic sidechain at the tip of loop L4 (such as UbcH5-F62). Correspondingly, a highly conserved proline residue on loop L2 of the RING/U-box type ligases (such as CHIP-P269) helps to position both hydrophobic and polar residues on this loop for interactions with E2 enzymes. This proline also directly interacts with the serine and alanine of the E2 S-P-A motif (which contains a third key proline residue) through its mainchain carbonyl group and through hydrophobic contacts respectively. It may be difficult to directly test the importance of these prolines by mutagenesis, as the corresponding mutants are likely to be improperly folded or insoluble in bacterial expression systems (data not shown).

The central hydrophobic interface is flanked by polar interactions contributed by sidechains from the E2-α1 helix and loop L7. A significant number of these determinants involve sidechain-mainchain interactions. Interestingly, several stacking interactions between arginine guanidinium groups and aromatic sidechains are present in the CHIP complexes, and likely play a role in the UbcH5:CNOT4 complex as well. In addition, an arginine on loop L2 of the ligase domains (R272 in CHIP) extends to interact with mainchain groups on loop L7 of the E2 enzymes. Overall these flanking interactions are less conserved among the E2:E3 interactions of known structure, and likely among E2:E3 complexes in general, than the central hydrophobic interaction surface. For example, no aromatic or hydrophobic residues corresponding to CHIP-F237 are present in c-Cbl or many other RING/U-box type ligases. Aromatic residues appear at this position in only a third of such ligase domains. Similarly, the flanking arginine (such as CHIP-R272) is conserved in only some U-boxes and RING domains, although there are often other polar residues with long sidechains at this position.

The relative importance of individual interactions in determining the overall affinity differs among E2:E3 complexes. For example, the CHIP-R272A and F237A mutants promote polyubiquitin formation by Ubc13 but do not interact productively with UbcH5 (Figure 4). Similarly, Ubc13 retains its interaction with CHIP even when M64, the hydrophobic residue on loop Ubc13-L4 that points into the hydrophobic pocket of the CHIP U-box, is mutated to alanine. This residue is a tyrosine or phenylalanine in approximately half of E2 enzymes, and hydrophobic in another 25%, but polar in the remaining E2 enzymes. This residue is somewhat less conserved than is the hydrophobic character of its binding pocket on the RING/U-box domain surface. This suggests that several other residues, such as the conserved prolines on E2-L4 and L7, can provide enough hydrophobic interaction area to support some E2:E3 interactions, depending on whether other supporting interactions are present.

CHIP binds 3- to 5-fold more strongly to uncharged Ubc13 than UbcH5a in isothermal titration calorimetric measurements, although both affinities are in the micromolar range ([22]; Z.X. and S.M., unpublished data). This suggests that the total binding energies are similar in the two complexes but are distributed differently among individual interactions in the binding interfaces. For example, a salt bridge is present between Ubc13-α1 and CHIP-L1 that has no counterpart in the UbcH5:CHIP complex (Figure 3B). The relative insensitivity of Ubc13-Uev1a to single mutations in the CHIP hydrophobic interface may also reflect the slightly higher affinity of this E2:E3 complex. It remains to be seen whether the affinity of CHIP for the ubiquitin-loaded forms of Ubc13 and UbcH5 are similar. We will also be investigating whether we can disrupt Ubc13:CHIP interactions by combining two Ubc13 mutations that, by themselves, do not disrupt interaction with CHIP.

A preponderance of hydrophobic and sidechain-mainchain interactions, rather than sidechain-sidechain interactions, promotes the close apposition of the interacting surfaces in the CHIP:UbcH5a and CHIP:Ubc13 complexes. This highlights the role that the avoidance of steric clashes may play in E2:E3 interaction selectivity. The S-P-A motif, which appears to be required for interaction with the CHIP U-box, provides an example of both negative steric selectivity and positive interaction selectivity, since the serine side chain forms a key hydrogen bond with a mainchain proline carbonyl group from the U-box. Substitution of the serine with an alanine, as in Ubc1, prevents the E2 enzyme from productively interacting with CHIP [18,20]. Other E2 enzymes that have bulky residues at positions equivalent to the S-P-A motif are also unable to interact with CHIP (Table 3).

In the Cbl:UbcH7 complex, a lysine residue replaces the serine, and the local conformations of the UbcH7-L7 and Cbl-L2 loops are different than those in the UbcH5a:CHIP complex, contributing to different polar interactions and accommodating the larger lysine side chain [30]. The N-terminal helix and loops L4 and L7 have slightly different conformations in UbcH7 than their counterparts in UbcH5a and Ubc13, possibly preventing them from orienting the relevant groups correctly to partner with the CHIP U-box. For example, the key E2-α1 arginine residue (such as UbcH5a-R5) interacts with a different loop L1 mainchain carbonyl in the Cbl:UbcH7 and CNOT4:UbcH5b complexes than in the CHIP complexes (Table 3). An inability to form this hydrogen bond may be the reason why the UbcH7-K96S mutant did not interact productively with CHIP (Figure 5B). Superimposition of UbcH7 onto the UbcH5a:CHIP U-box structure also suggests that UbcH7-R6, the equivalent of UbcH5a-R5, would clash with CHIP-F227, while Cbl has an alanine residue at the position corresponding to CHIP-F227 (data not shown). Interestingly, Cbl has been shown to interact with both UbcH7 and UbcH5 [35]. In addition, Cbl engages E2 enzymes not only through the RING domain but also through a secondary binding site between a helical region and the N-terminal helix of the E2 enzymes [30]. This may compensate for the absence of an interaction equivalent to the UbcH5a-R5: CHIP-F227 interaction.

The number and similarity of some of the specificity determinants among the known E2:E3 complexes suggests that many E3 ligases interact with more than one type of E2 enzyme. This has several potential functional consequences. One possibility is that an E3 ligase interacts with different E2 enzymes in different intracellular contexts. We found that CHIP interacts *in vitro *with a representative Class III E2 enzyme, Ube2e2. Ube2e2 and its close homologues Ube2e1 and Ube2e3 undergo nuclear import after they are charged with ubiquitin [33]. CHIP is partially localized to the nucleus [36], suggesting that CHIP may be a partner for these Class III E2 enzymes in the ubiquitination of nuclearly localized substrates.

Another rationale for the ability of an E3 ligase to interact with multiple E2 enzymes is specific to Ubc13. Ubc13 is the only E2 enzyme that exclusively synthesizes K63-linked polyubiquitin chains, in a manner dependent on its heterodimerization with a UEV (Ubiquitin E2 variant) protein, such as Uev1a, Uev1b or Mms2 [25,37]. UEVs have an E2-like fold but lack a catalytic cysteine. Instead, they bind ubiquitin noncovalently and position K63 of the noncovalently bound ubiquitin for conjugation to the thioester-linked ubiquitin on Ubc13, thus forming K63-linked polyubiquitin [38,39]. Ubc13/UEV heterodimers may be unable to directly ubiquitinate some substrates, instead forming free K63-linked chains [24,40]. Accumulating evidence, however, suggests that Ubc13/UEV heterodimers may also bind to a substrate-conjugated ubiquitin moiety through the UEV subunit [41]. This could allow Ubc13 to attach additional ubiquitins to the initial ubiquitin, forming a substrate-conjugated K63-linked chain. Ubc13/UEV heterodimers may thus rely on other E2 enzymes, which must be compatible with the E3 ligase participating in the ubiquitination reaction, to "preubiquitinate" a given substrate. Ubc13-Uev1a and Ubc13-Mms2 participate in distinct intracellular processes with different intracellular localization [42]. Intracellular localization may further limit which preubiquitinating E2 enzymes are available. If the preubiquitinating E2 enzyme is itself capable of polyubiquitination, the relative affinities of the preubiquitinating E2 enzyme and Ubc13 for the E3 ligase may also regulate the type of polyubiquitin chain that is added to the substrate, as both E2 enzymes may compete for binding to the E3 ligase.

## Conclusion

In this article, we describe the structural basis for the interaction of the U-box of CHIP with its cognate E2 enzyme UbcH5a. Binding of CHIP to UbcH5a and Ubc13 enzymes is mediated by a similar set of interacting groups, which resemble those observed in other structurally characterized RING:E2 enzyme complexes. The CHIP:UbcH5a complex forms through close packing of complementary hydrophobic surfaces, surrounded by polar interactions. The S-P-A motif, located on loop L7 of UbcH5a and Ubc13 acts as an important specificity determinant; E2 enzymes lacking this motif are not cognate enzymes for CHIP. Based on the conservation of the S-P-A motif, the Class III E2 enzyme Ube2e2 and its homologues Ube2e1 and Ube2e3 are also cognate E2 enzymes for CHIP. CHIP may have to interact sequentially other E2 enzymes and Ubc13-Uev1a in order to attach K63-linked polyubiquitin chains on substrates, as CHIP only stimulates the formation of free K63-polyubiquitin by Ubc13-Uev1a. This provides one functional rationale for the ability of CHIP and other E3 ligases to interact with multiple E2 enzymes.

## Methods

### Protein expression and purification

For crystallization trials, Zebrafish (*Brachydanio rerio*) CHIP residues 204–284 (encompassing the U-box) and UbcH5a were cloned into pHis||2 vector [43] and expressed as His-tagged fusion proteins in *E. coli *Rosetta2(DE3) cells (Novagen) at 20°C for 16 hours after induction with 0.5 mM IPTG. The proteins were purified by Nickel affinity chromatography in 50 mM Sodium Phosphate pH 7.7, and 300 mM NaCl. After overnight cleavage with Tobacco Etch Virus (TEV) protease, cleaved His-tags were removed using a second round of Nickel-affinity chromatography. Additional purification was performed by Superdex 75 gel filtration column in 50 mM Tris pH 7.6, 150 mM NaCl.

For *in vitro *ubiquitination assays, human CHIP, Ubc13, and UbcH7 were cloned into the pHis||2 vector and expressed as above. Submilligram quantities of His-tagged CHIP were purified using a protein miniprep kit (His-Spin kit, Zymo Research) and used for subsequent assays without removal of the His-tags. Larger quantities of His-tagged CHIP, Ubc13 and UbcH7 were purified by Nickel affinity chromatography as described above, except that buffers were at pH 7.4 (CHIP and Ubc13) or pH 7.8 (UbcH7). Human UbcH5b and Uev1a were cloned into pGST||2 vector, expressed as glutathione S-transferase (GST) fusion proteins, and purified by glutathione-sepharose chromatography in 150 mM NaCl and 50 mM sodium phosphate, pH 7.8. GST tags were removed by overnight cleavage with TEV protease during dialysis in 100 mM NaCl, 50 mM sodium phosphate pH 7.0, 5 mM 2-mercaptoethanol, followed by a second pass over the glutathione resin.

Site-directed mutations were introduced into full-length His-tagged human CHIP or the E2 enzymes using the QuickChange mutagenesis kit (Stratagene) and verified by sequencing. His-tagged CHIP mutants were bacterially expressed similarly to the wild type protein and purified at small scales using the His-spin protein miniprep kit (Zymo Research). E2 enzyme mutants were expressed as GST-fusions and purified like GST-tagged Uev1a with appropriately adjusted buffer pH values. Expression and purity of the mutants were quantified by SDS-PAGE.

### Crystallization, structure determination and refinement

Zebrafish CHIP U-box and UbcH5a were dialyzed into crystallization buffer (50 mM NaCl, 20 mM Tris-HCl (pH7.4), 2 mM DTT), combined in a 1:1 ratio and incubated at 4°C for 3 hours. The complex was crystallized at 20 mg/mL by hanging-drop vapor-diffusion over 1.65 M Ammonium Sulfate, 90 mM Bis-Tris (pH 6.7) at 20°C. Oblong crystals grew to a size of 1 × 0.2 × 0.2 mm^3 ^after 4–5 days. Crystals were cryoprotected in reservoir solution supplemented with 20% glycerol and frozen in liquid nitrogen. Native data sets were collected at Advanced Light Source beamline 4.2.2., Lawrence Berkeley National Laboratory. Data were indexed, integrated and scaled using D*TREK [44]. Molecular replacement trials in Phaser [45] and CNS [46] using a combination of previously solved structures of the Zebrafish CHIP U-box [PDB:2F42] and human UbcH5b [PDB:2ESK] were successful. After model rebuilding using COOT [47], refinement against a 2.9 Å dataset in CNS resulted in a final model with R = 0.240, R_free _= 0.272, and no Ramachandran violations as judged by Procheck [48]. Crystallographic data collection and refinement statistics are summarized in Table 1. Molecular graphics were generated using PyMol 0.99 [49].

### *In vitro *ubiquitination assays

Ubiquitination assays subject to subsequent western blotting were performed following the protocol of Murata [50] in 50 μl reaction buffer (50 mM Tris pH 7.6, 4 mM ATP, 2 mM MgCl_2_, 1 mM DTT). Reactions containing 2 μg CHIP, 100 ng E1 enzyme (Boston Biochem), 1 μM E2 enzyme and 6 μg bovine ubiquitin (Sigma) were incubated at 30°C for 2 hours and terminated by addition of SDS-PAGE sample buffer. Western blotting with HRP-conjugated anti-ubiquitin (Santa Cruz Biotechnology) was used to identify ubiquitinated species. Selected blots were probed with anti-CHIP (Calbiochem) and HRP-conjugated goat anti-rabbit antibody.

ELISA-based ubiquitination assays were carried out by a similar protocol as described by Huang and coworkers [51]. Reactions were performed in Ni-NTA coated microplates (Pierce His-grab) in 100 μL reaction buffer (50 mM Tris pH 7.6, 50 mM Imidazole, 3.3 mM ATP, 25 mM MgCl_2_, 1 mM DTT). Plates were preblocked with Protein free Phosphate Buffered Saline (PBS) blocking buffer (Pierce) to prevent nonspecific binding. Reactions contained 5 μg His-tagged or untagged CHIP, 50 ng E1 enzyme (Boston Biochem), 1 μM untagged E2 enzyme, 1 μg Flag-tagged ubiquitin (Boston Biochem) and, in select experiments, 0.2 μg His-tagged ubiquitin (Boston Biochem). Reactions were incubated at 30°C for 2 hours and washed 3 times with 1 × PBS. Anti-Flag antibody and Horseradish Peroxidase (HRP)-conjugated anti-mouse secondary antibody were used to detect Flag-ubiquitinated His-tagged CHIP or mixed His-tagged/Flag-tagged polyubiquitin retained on the Ni-NTA coated plates. Unbound antibodies were removed with 3 additional PBS washes followed by addition of 100 μL luminol substrate (Pierce). Luminescence was measured on a Veritas microplate luminometer (Turner Bioystems). Data from 3–6 independent trials were averaged.

## Abbreviations

E1: Ubiquitin activating enzyme; E2: Ubiquitin conjugating enzyme: E3: Ubiquitin ligase; CHIP: C-terminus of Hsc70 Interacting Protein; TPR: Tetratricopeptide repeat; NTA: Nitrilotriacetic acid; UEV: Ubiquitin E2 variant; IPTG: Isopropyl β-D-1-thiogalactopyranoside; DTT: Diothiothreitol; GST: Glutathione S-Transferase; ELISA: Enzyme-Linked ImmunoSorbent Assay.

## Authors' contributions

XZ and SM crystallized the complex and solved and refined the crystal structure. EK designed the ELISA-based ubiquitination assays. EK, XZ, KID and MB cloned and purified CHIP, CHIP U-box and E2 enzymes and carried out in vitro ubiquitination assays. JCN collected and processed crystallographic data and assisted in solving the structure. SM was additionally involved in designing and overseeing the study. XZ, EK and SM prepared the manuscript. All authors have read and approved the final manuscript.
